# Synthesis‐Dependent Grain Boundary Density Governs Low‐Temperature Lithium Diffusion in Single‐Crystal NCM Cathodes

**DOI:** 10.1002/advs.77448

**Published:** 2026-08-31

**Authors:** Yeong Beom Kim, Jaewon Lee, Jihong Park, Seunghyeon Chae, Dohyung Kim, Gigap Han, Hyeongi Song, Jihye Jang, Jihoon Choi, Sang Mun Jeong, Yun Chan Kang, Gi Dae Park

**Affiliations:** ^1^ Department of Materials Science and Engineering Korea University Seoul Seongbuk‐gu Republic of Korea; ^2^ Department of Advanced Materials Engineering Chungbuk National University, Chungbuk Seowon‐gu Republic of Korea; ^3^ Department of Urban, Energy, and Environmental Engineering Chungbuk National University, Chungbuk Seowon‐gu Republic of Korea; ^4^ SK On R&D Center Daejeon Republic of Korea; ^5^ Department of Chemical Engineering Chungbuk National University, Chungbuk Seowon‐gu Republic of Korea; ^6^ Advanced Energy Research Institute Chungbuk National University, Chungbuk Seowon‐gu Republic of Korea

**Keywords:** grain boundary effects, low‐temperature lithium diffusion, mid‐nickel NCM cathodes, single‐crystal cathodes, spray pyrolysis

## Abstract

Mid‐nickel LiNi_0.6_Co_0.1_Mn_0.3_O_2_ (NCM613) single‐crystal cathodes were synthesized via co‐precipitation and spray pyrolysis routes and systematically compared to elucidate the relationship between synthesis‐driven single‐crystallization efficiency, internal grain boundary characteristics, and electrochemical performance. Although both materials exhibit comparable particle sizes and chemical compositions, pronounced differences in internal grain boundary density were observed depending on the synthesis route. The spray pyrolysis–derived NCM613 forms a highly integrated single‐crystal‐like structure with a substantially reduced density of internal grain boundaries compared to its co‐precipitation‐derived counterpart. Electrochemical and kinetic analyses reveal that residual internal grain boundaries disrupt continuous Li‐ion diffusion, resulting in pronounced performance deterioration that becomes particularly severe under low‐temperature operation. At −10°C, increased grain boundary density induces significant transport heterogeneity and persistent surface microcracking, ultimately accelerating electrochemical degradation.

## Graphical abstract

The relationship between synthesis‐dependent crystallization behavior, internal grain‐boundary density, and intrinsic lithium diffusion kinetics in single‐crystal mid‐Ni NCM cathodes is elucidated, providing practical design insights for advanced cathode materials.

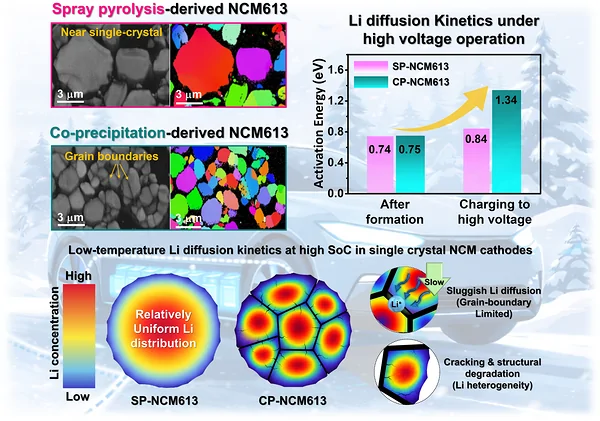

## 1 Introduction

Single‐crystal LiNi_1−x−y_Co_x_Mn_y_O_2_ (NCM) cathodes have emerged as promising candidates for next‐generation lithium‐ion batteries, owing to their high energy density and improved cycling durability [1−6]. In contrast to conventional polycrystalline cathodes, micron‐scale primary particles intrinsically exhibit lower specific surface areas and superior mechanical robustness, which effectively mitigate microcrack formation during repeated electrochemical cycling [7, 8]. However, the large particle size of single‐crystal NCM inevitably leads to extended Li‐ion diffusion pathways, resulting in sluggish reaction kinetics and inferior rate capability. Such kinetic limitations become particularly critical at low‐temperatures, where lithium‐ion transport is significantly hindered. Considering that practical lithium‐ion batteries are frequently required to operate under low‐temperature conditions, understanding and improving the low‐temperature behavior of cathode materials is highly desirable. Nevertheless, the electrochemical behavior of single‐crystal NCM cathodes under such conditions remains largely unexplored.

Particle size and morphological characteristics strongly influence Li‐ion diffusion pathways and surface reaction kinetics, thereby affecting rate capability as well as structural degradation and capacity decay during prolonged cycling [9, 10]. Notably, so‐called “single‐crystal” NCM particles are rarely ideal single‐crystals in a strict crystallographic sense. Instead, individual particles frequently consist of a small number of crystallographic domains, placing them closer to quasi‐single‐crystalline architectures [7, 11]. Such residual grain boundaries are not merely structural imperfections; they actively serve as origins for intergranular cracking and accelerate electrochemically induced phase and structural degradation [12, 13]. Previous studies have shown that even a limited number of grain boundaries can significantly compromise the long‐term stability of quasi‐single‐crystal NCM cathodes, underscoring the importance of precise control over crystallization and internal domain structures [14]. This issue becomes more critical under low‐temperature conditions, where sluggish Li‐ion diffusion amplifies transport heterogeneity at internal grain boundaries. In particular, the correlation between internal grain boundary characteristics and Li‐ion transport behavior under low‐temperature conditions remains poorly understood.

Since internal grain boundary characteristics are largely governed by crystallization behavior during synthesis, precise control over the synthetic process is essential for realizing well‐defined single‐crystal architectures. Conventional single‐crystal NCM cathodes are typically synthesized via high‐temperature calcination of co‐precipitated precursors [15, 16], where the initial microstructure is largely preserved, rendering the formation of truly single‐crystalline particles intrinsically challenging [17, 18]. Therefore, developing synthesis strategies that effectively suppress residual grain boundaries is essential for rational single‐crystal design. Among various approaches, spray pyrolysis offers a distinct advantage by directly generating compositionally homogeneous precursor particles through rapid droplet‐to‐particle conversion [19]. The uniform incorporation of lithium species shortens the Li‐transition metal interdiffusion length during calcination, facilitating efficient single‐crystal growth under milder conditions [20].

Given their commercial relevance and favorable thermal stability for single‐crystal growth, mid‐Ni NCM compositions (50% ≤ Ni ≤ 60%) provide a suitable platform to probe synthesis‐structure‐property relationships [21, 22]. In this study, single‐crystal LiNi_0.6_Co_0.1_Mn_0.3_O_2_ (NCM613) was synthesized via co‐precipitation and spray pyrolysis, and the resulting structural and electrochemical properties were systematically compared. The two samples exhibit pronounced differences in internal grain boundary characteristics. Owing to the efficient single‐crystallization of spray pyrolysis precursors, the resulting NCM613 (SP‐NCM613) displays a highly integrated single‐crystal‐like structure with a markedly reduced density of internal grain boundaries compared to its co‐precipitation‐derived counterpart (denoted as CP‐NCM613). Temperature‐dependent electrochemical and kinetic analyses further reveal that grain boundary density becomes a dominant limitation on Li‐ion diffusion at low‐ temperatures. Under these kinetically constrained conditions, abundant grain boundaries in CP‐NCM613 disrupt continuous diffusion pathways, resulting in irreversible surface gliding and microcrack formation.

## 2 Results and Discussion

**FIGURE 1:**
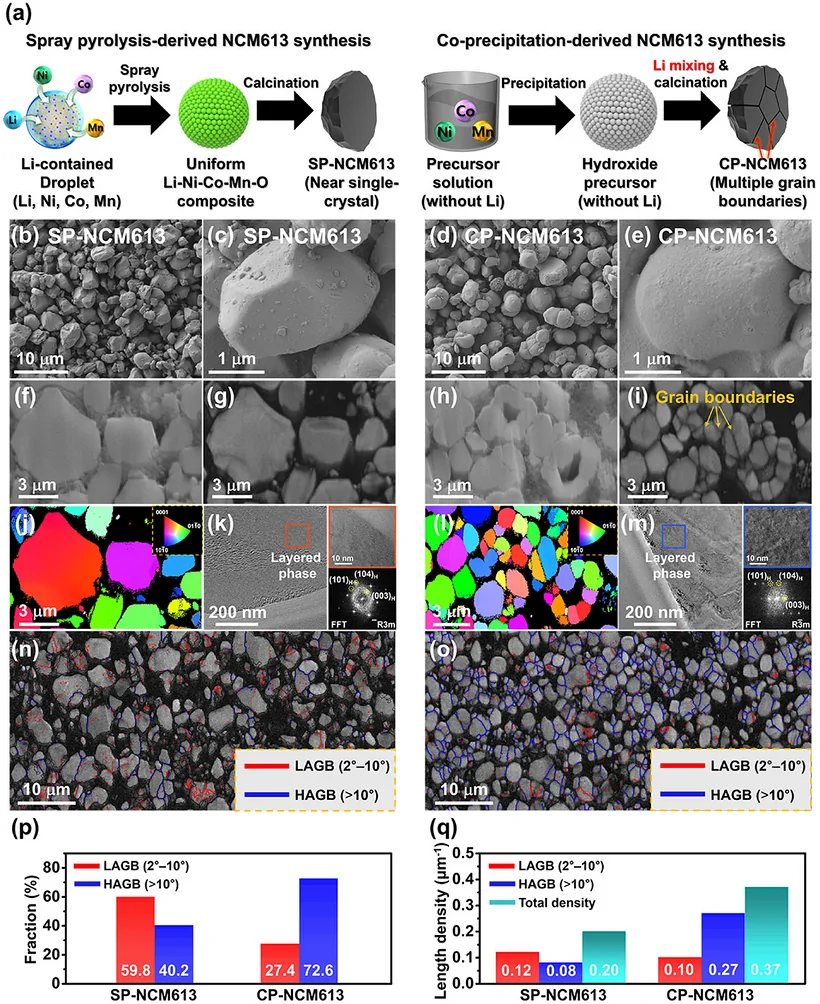
a) Schematic illustration of the synthesis routes for single‐crystal NCM613 cathodes via spray pyrolysis and co‐precipitation. SEM images of b,c) SP‐NCM613, and d,e) CP‐NCM613, Cross‐sectional SEM images and band contrast EBSD maps of f,g) SP‐NCM613 and h,i) CP‐NCM613. EBSD orientation maps of j) SP‐NCM613 and l) CP‐NCM613. HR‐TEM images and corresponding FFT patterns of k) SP‐NCM613 and m) CP‐NCM613. Grain boundary maps of n) SP‐NCM613 and o) CP‐NCM613. p) Fractions of low‐angle grain boundary (LAGB) and high‐angle grain boundary (HAGB). q) Grain boundary length densities of LAGB and HAGB.

The synthesis mechanisms via different routes are illustrated in Figure 1a. In the spray pyrolysis process, all metal salts, including the lithium source, are dissolved in distilled water and incorporated into each aerosol droplet. As a result, the precursor particles contain homogeneously distributed Li, Ni, Co, and Mn species. The uniform lithium distribution shortens the interdiffusion length during calcination, thereby facilitating efficient crystallization and leading to particles with minimal grain boundaries [23−26]. In contrast, hydroxide precursors synthesized via co‐precipitation do not contain lithium and require subsequent mixing with a lithium source prior to calcination. During heat treatment, lithium must diffuse inward from the particle surface, and the pre‐formed crystallographic framework of the precursor can impede complete single‐crystallization, resulting in residual grain boundaries within the particles [27, 28]. To further improve electronic conductivity and mitigate parasitic surface reactions, the synthesized cathode materials were sequentially surface‐modified using Co‐ and W‐containing precursors through post‐heat‐treatment processes. All synthesis procedures were conducted at SK On, and specific processing conditions are not provided due to confidentiality constraints.

The characterization results of the precursor materials (denoted as SP‐precursor and CP‐precursor), including their morphology, crystallinity, and particle‐size distribution, are provided in Figure S1 and described in the Supplementary Information. Although the precursor characteristics differed depending on the synthesis route, the resulting micron‐sized NCM613 cathodes exhibited comparable particle size distributions and chemical compositions, while the internal grain boundary density remained the most pronounced microstructural difference. The resulting NCM613 morphologies were subsequently examined. SEM images (Figure 1b–e) show that both samples consist of single‐crystal particles with comparable sizes in the few‐micrometer range, which is further supported by similar D50 values of ∼4.31 µm (Figure S2a,b). Notably, CP‐NCM613 exhibits a more spherical morphology than SP‐NCM613, reflecting morphological inheritance from the spherical co‐precipitated precursors. The Brunauer‐Emmett‐Teller (BET) specific surface areas of the two samples were also comparable (Figure S2c,d), consistent with their similar particle size distributions. Furthermore, cross‐sectional SEM analysis of the ion‐milled electrodes revealed comparable electrode densities of 3.32 and 3.23 g cm^−3^, corresponding to electrode porosities of 19.6% and 21.6% for SP‐NCM613 and CP‐NCM613, respectively (Figure S3). Despite the slight difference in particle morphology, both samples exhibited comparable electrode densities and porosities, indicating similar electrode packing characteristics. Therefore, the influence of particle morphology and electrode structure on the electrochemical performance is considered to be minimal. To probe internal grain boundary characteristics, cross‐section polishing followed by electron backscatter diffraction (EBSD) analysis was performed. As shown in Figure S4, in contrast to the cross‐sectional SEM images, the band contrast and EBSD inverse pole figure (IPF) maps enable more distinct visualization of internal defects, such as grain boundaries, within the particles [29]. High‐magnification images are presented in Figure 1f–i. Interestingly, despite identical calcination temperatures applied to both samples, SP‐NCM613 (Figure 1g,j) exhibits fewer internal grain boundaries and a microstructure closer to a single‐crystal nature compared to CP‐NCM613 (Figure 1i,l). Based on these observations, the two samples with distinct internal grain boundary densities were systematically compared to elucidate their influence on electrochemical behavior. The elemental mapping images in Figure S4d,h reveal a uniform distribution of the constituent elements in NCM613. Although slight agglomeration of the surface modification species (Co and W) is observed, these species are broadly distributed throughout the electrode, suggesting their effective contribution to the surface modification process. Moreover, high‐resolution TEM images and the corresponding FFT patterns (Figure 1k,m) confirm that both samples exhibit a well‐defined layered structure with an R3̅m space group [30, 31]. To provide a quantitative comparison of the internal grain boundary characteristics, additional EBSD analysis was performed based on the raw orientation data. Grain boundary maps were generated to determine the grain boundary length density and grain boundary character distribution (Figure 1n–q). In EBSD analysis, grain boundaries are commonly classified according to the misorientation angle between neighboring domains into low‐angle grain boundary (LAGB, 2°–10°) and high‐angle grain boundary (HAGB, >10°) [32]. LAGBs are generally associated with arrays of dislocations, whereas HAGBs represent crystallographic interfaces with larger misorientation angles [33]. Accordingly, the grain boundary character distribution provides quantitative information regarding the internal crystallographic characteristics of individual particles. In the grain boundary maps, LAGBs and HAGBs are represented by red and blue lines, respectively. As shown in Figure 1n,o, CP‐NCM613 exhibits a substantially higher overall grain boundary density than SP‐NCM613. Furthermore, the grain boundary character distribution (Figure 1p) reveals that LAGBs are predominant in SP‐NCM613, whereas CP‐NCM613 exhibits a significantly higher fraction of HAGBs. Quantitative analysis of the grain boundary length density (Figure 1q) further demonstrates that the two samples possess comparable LAGB densities, while the HAGB density of CP‐NCM613 is markedly higher than that of SP‐NCM613. These results indicate that CP‐NCM613 contains a greater density of crystallographically discontinuous internal grain boundaries, whereas SP‐NCM613 possesses a more crystallographically coherent internal structure.

**FIGURE 2:**
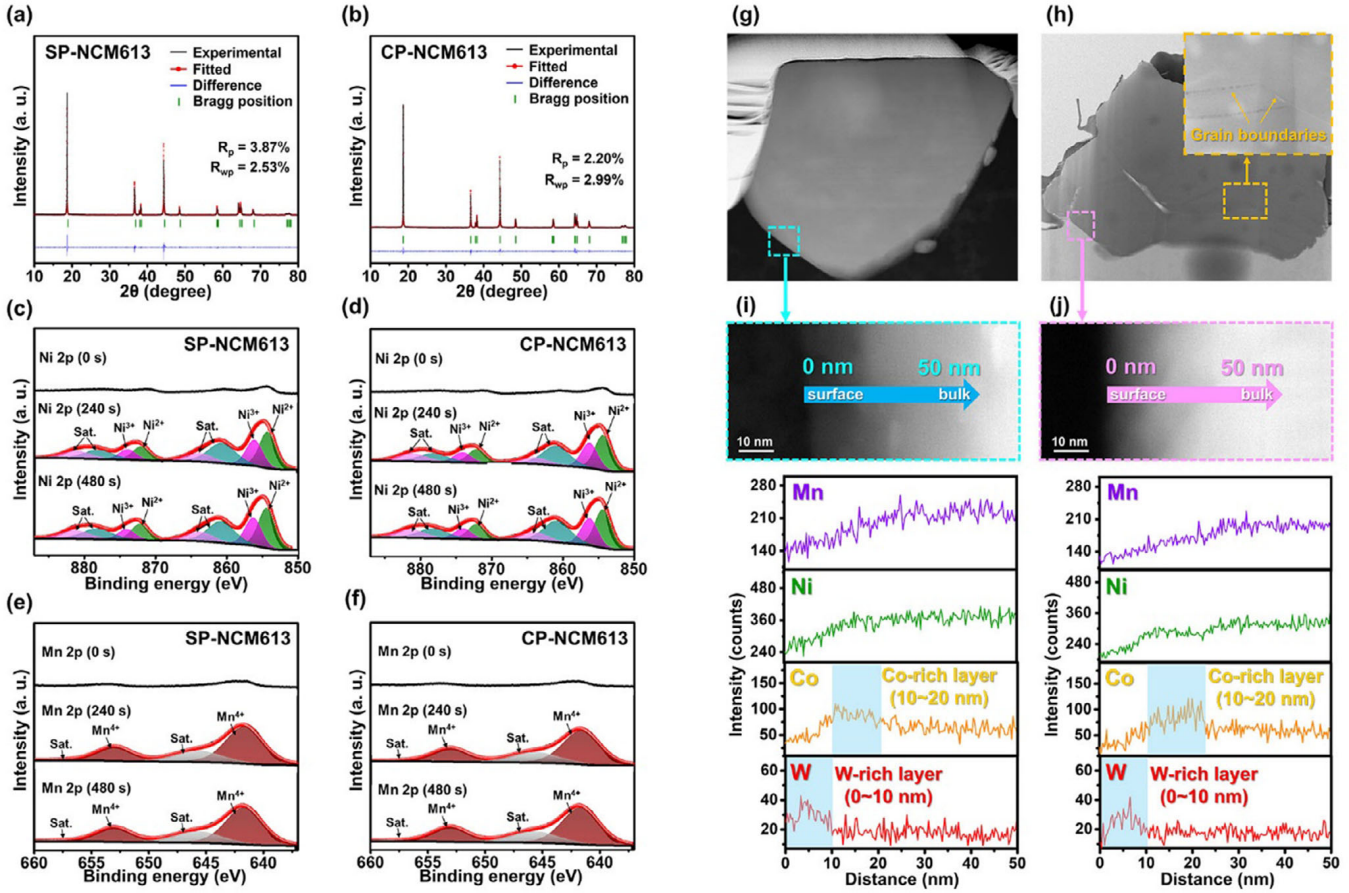
Rietveld refinement of XRD patterns for a) SP‐NCM613 and b) CP‐NCM613. XPS depth profiles of Ni 2p for c) SP‐NCM613 and d) CP‐NCM613; Mn 2p for e) SP‐NCM613 and f) CP‐NCM613, obtained after sputtering for 240 and 480 s. HAADF‐STEM images of g) SP‐NCM613 and h) CP‐NCM613. STEM‐EDS line‐scan profiles of i) SP‐NCM613 and j) CP‐NCM613 showing the elemental distributions across the surface‐modified regions.

To examine the crystal structures and chemical states, X‐ray diffraction (XRD) with Rietveld refinement and depth‐profiling X‐ray photoelectron spectroscopy (XPS) analyses were performed (Figure 2). Both samples exhibit a well‐defined layered structure with the R3̅m space group (Figure 2a,b), and comparable lattice parameters and Li/Ni cation mixing values (Table S1). SP‐NCM613 exhibits a slightly higher I_(003)_/I_(104)_ ratio, suggesting a more ordered layered structure. Depth‐profiling XPS analysis was conducted to clarify the distribution of coating components and the bulk chemical states (Figure 2c–f and Figure S5). The Ni 2p and Mn 2p spectra for both samples exhibit increased signal intensities after Ar^+^ sputtering, while maintaining binding energies characteristic of layered NCM613, confirming that the bulk chemical states remain unchanged [34−38]. Similarly, the Co 2p spectra (Figure S5a,b) exhibit enhanced signal intensities after Ar^+^ sputtering. This behavior can be attributed to the presence of a Co‐enriched subsurface region beneath the outermost surface, where the relative contribution from Co‐containing species increases as the surface layer is gradually removed during sputtering [39, 40]. In contrast, the W 4f spectra (Figure S5c,d) show a gradual decrease in intensity with increasing sputtering depth [41, 42], indicating that W is preferentially enriched near the outermost surface compared with Co. The chemical compositions of the precursor and final cathode materials were analyzed by ICP‐OES. ICP analysis confirmed that the elemental compositions of both precursors (Table S2) were consistent with the target composition of NCM613 (Ni:Co:Mn = 6:1:3). In particular, the spray pyrolysis‐derived precursor exhibited a Li/TM molar ratio of approximately 1.07, confirming the successful incorporation of lithium into the precursor during the spray pyrolysis process. ICP analysis of the final surface‐modified cathode materials (Table S3) confirmed comparable Ni:Co:Mn compositions for both samples, along with similar contents of the introduced surface‐modifying elements. Based on the ICP results, the amounts of Co and W surface modifiers were estimated to be approximately 2.5 and 0.5 wt.%, respectively. To investigate the elemental distribution across the surface‐modified region, complementary STEM‐EDS and STEM‐EELS analyses were conducted, as shown in Figure 2g−j and Figure S6. The HAADF‐STEM images of both samples (Figure 2g,h) also revealed distinct differences in their internal structures. Internal grain boundaries were not readily discernible in SP‐NCM613, whereas CP‐NCM613 exhibited numerous internal grain boundaries separating multiple subgrains within individual particles. Furthermore, STEM‐EDS line‐scan analysis was performed from the particle surface toward the bulk over a distance of approximately 50 nm (Figure 2i,j). A W‐enriched outermost surface region followed by a Co‐enriched subsurface region was identified, after which the elemental compositions gradually converged to those of the NCM613 bulk. The compositional gradient extended approximately 20 nm from the particle surface. Additionally, STEM‐EELS line‐scan analysis was performed from the particle surface toward the bulk (Figure S6) to further examine the local compositional variation. Consistent with the STEM‐EDS results, the EELS spectra also exhibited a relatively enhanced Co signal within the near‐surface region (∼10–20 nm), whereas the Mn and Ni signals gradually increased toward the bulk before reaching stable intensity levels. These results indicate that the sequential surface modification produced W‐enriched outer and Co‐enriched subsurface regions with gradual compositional transitions, rather than discrete WO_3_ and LiCoO_2_ coating layers with well‐defined interfaces. This interpretation is also consistent with the depth‐profile XPS results. Overall, both samples possess nearly identical crystal structures, chemical environments, particle sizes, and compositions. The only discernible difference lies in the internal grain boundary density within individual particles. Therefore, this sample pair provides an ideal platform to clarify the intrinsic impact of residual grain boundaries on electrochemical performance.

**FIGURE 3:**
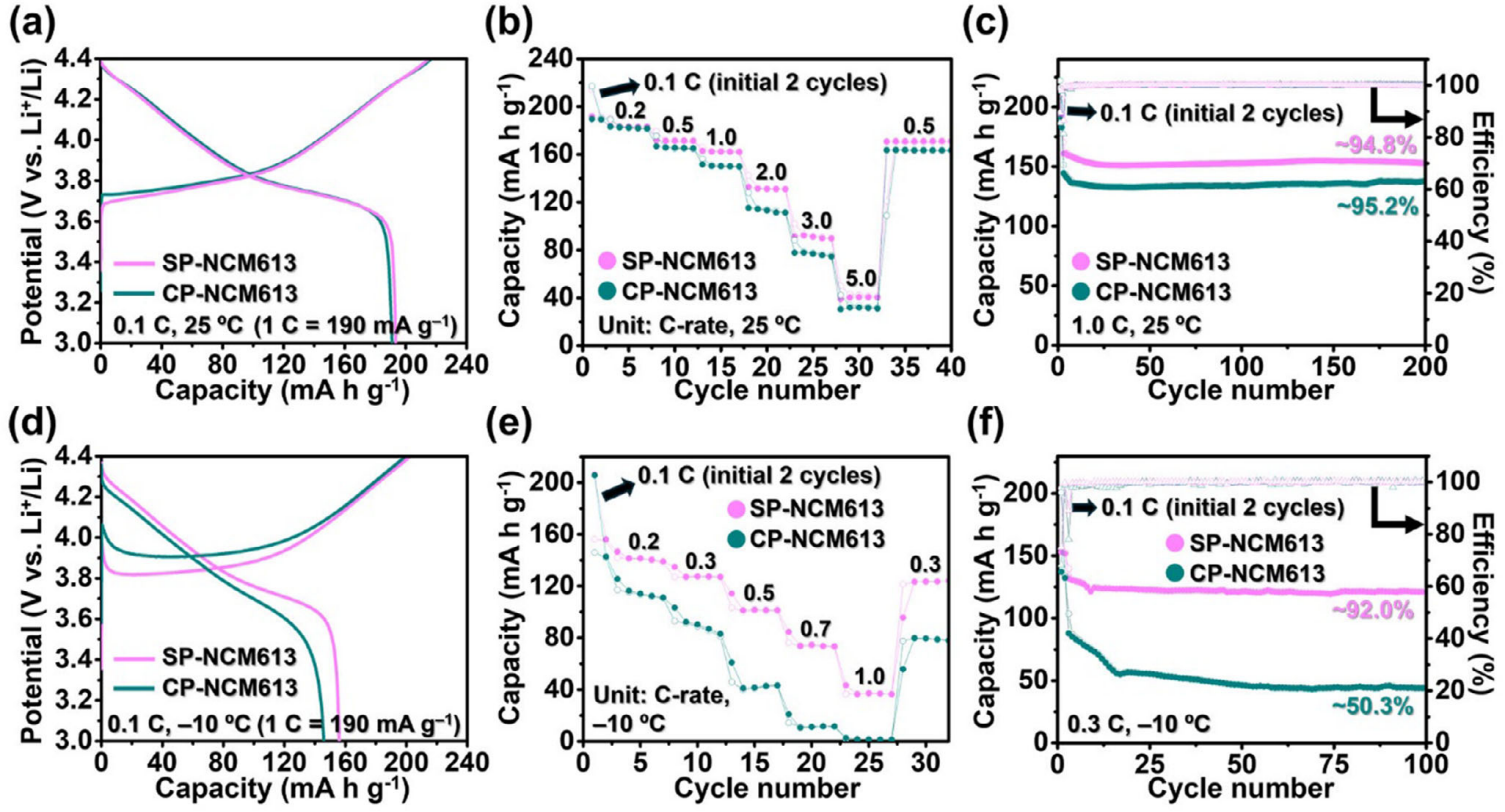
Half‐cell electrochemical performances of two cathodes at 25°C: a) voltage profiles during the initial cycle at 0.1 C‐rate, b) rate performances, and c) cycle performances at 1.0 C‐rate after two activation cycles at 0.1 C‐rate. Half‐cell electrochemical performances at −10°C: d) voltage profiles during the initial cycle at 0.1 C‐rate, e) rate performances, and f) cycle performances at 0.3 C‐rate after two activation cycles at 0.1 C‐rate.

Figure 3 demonstrates the electrochemical properties of two NCM613 cathodes. To evaluate the practical electrochemical behavior of single‐crystal cathodes, all measurements were conducted with a high‐voltage cutoff of 4.4 V and a high areal cathode loading of 12 mg cm^−2^. At 25°C (Figure 3a), SP‐NCM613 and CP‐NCM613 deliver initial discharge capacities of 193.5 and 191.0 mA h g^−1^ (0.1 C), with initial Coulombic efficiencies of 89.6% and 87.6%, respectively. To enable reliable evaluation of micron‐sized single‐crystal NCM613 cathodes under practical operating conditions, a Co/W‐based surface modification strategy was employed to improve interfacial kinetics and surface stability. As shown in Figure S7, the pristine NCM613 cathode (prepared via the spray pyrolysis route) without surface modification delivered an initial discharge capacity of 184.7 mA h g^−1^. After Co‐based surface modification, the initial discharge capacity increased to 190.1 mA h g^−1^, which can be attributed to the improved interfacial kinetics provided by the Co‐rich surface region [43]. Furthermore, the final W/Co dual surface‐modified SP‐NCM613 exhibited further improvement in electrochemical performance, which may be associated with the enhanced surface stability and reduced interfacial degradation provided by the W‐rich surface region [44]. In rate tests at 25°C (Figure 3b), both samples exhibit capacity decay above 3 C, reflecting the intrinsic kinetic limitations of micron‐sized primary particles [3, 45]. Nevertheless, SP‐NCM613 consistently delivers higher capacities across the high‐rate region, indicating that internal grain boundary density influences rate capability. At 1 C cycling (Figure 3c), both cathodes show stable performance over 200 cycles (94.8% and 95.2% retention for SP‐NCM613 and CP‐NCM613, respectively), while SP‐NCM613 maintains higher absolute discharge capacities throughout. In contrast, the performance difference becomes pronounced at −10°C. Both samples display voltage overshoot during initial charging (Figure 3d), indicative of sluggish kinetics [46]; however, CP‐NCM613 suffers substantially greater polarization, resulting in lower capacities and ICE. The capacities of both cathodes are reduced compared with those at room temperature, whereas the current density was maintained at the same C‐rate to ensure experimental consistency (1 C = 190 mA g^−1^, identical to that used at room temperature). Rate performance at −10°C (Figure 3e,f) further highlights this disparity: CP‐NCM613 exhibits severe capacity decay above 0.3 C, whereas SP‐NCM613 retains 127 mA h g^−1^ at 0.3 C and maintains 92.0% capacity after 100 cycles. Consistently, dQ/dV analysis (Figures S8 and S9) reveals comparable polarization evolution at 25°C but markedly aggravated peak broadening and voltage shifts for CP‐NCM613 under low‐temperature operation, evidencing deteriorated reaction kinetics. It is worth noting that the shift from the second to the fourth cycle mainly results from the increase in current density from 0.1 C to higher rates (0.3 or 1.0 C), which leads to enhanced polarization. Although the electrochemical performance of CP‐NCM613 may appear relatively lower than previously reported NCM cathodes, direct comparison is challenging because of the substantial differences in evaluation conditions among studies. As summarized in Table S4, previously reported single‐crystal NCM cathodes were generally evaluated under different electrode configurations, often involving lower electrode loading and electrode density. Such differences can significantly influence the measured electrochemical performance, even for cathode materials with similar compositions, due to variations in transport limitations and polarization behavior. Notably, the low‐temperature behavior of micron‐sized NCM cathodes evaluated under practical electrode conditions with high electrode loading and high electrode density remains largely unexplored. Therefore, the performance of CP‐NCM613 should be interpreted considering the intrinsic kinetic limitations associated with micron‐sized cathodes containing internal grain boundaries under practical operating conditions. Furthermore, full cell tests employing a commercial graphite anode were conducted to evaluate the practical applicability of the cathode materials. Prior to full cell evaluation, the electrochemical performance of the graphite anode was examined in half cells. At room temperature (Figure S10a), the graphite anode delivered a capacity of 335.5 mA h g^−1^ at a current density of 0.035 A g^−1^, indicating typical behavior. In contrast, at −10°C (Figure S10b), the capacity decreased significantly even at the same current density. Although reducing the current density by half led to a partial recovery in capacity, the overall value remained substantially lower than that at room temperature. These results suggest that the graphite anode suffers from sluggish reaction kinetics under low‐temperature conditions, indicating that its contribution becomes a critical limiting factor in low‐temperature full cell operation. As shown in Figure S11a,b, the SP‐NCM613‐based full cell retains superior electrochemical performance at room temperature, consistent with the half‐cell results (Figure 3a,c). Based on practical cell design, the N/P ratio of the electrodes was controlled to be approximately 1.1 under room‐temperature conditions. However, at low‐temperature, the reduced capacities of both electrodes induce a shift in the effective N/P ratio. As a result, when the same full cell is operated at −10°C (Figure S11c,d), it becomes difficult to achieve the nominal capacity. Nevertheless, the full cell still maintains excellent cycling stability under these conditions. Therefore, not only cathode optimization but also improving the low‐temperature performance of graphite anodes is essential for achieving reliable full cell operation, as also highlighted in previous studies [47, 48].

To further elucidate the temperature‐dependent kinetic behavior, in situ EIS measurements coupled with DRT analysis were performed during the first cycle. The detailed DRT profiles at 25°C are provided in Figure S12. In the DRT profile, individual relaxation times are associated with distinct electrochemical processes, enabling the deconvolution of kinetic contributions. Seven major relaxation peaks are consistently observed across all DRT spectra during both charge and discharge processes. Specifically, the peak in the 10^−5^–10^−4^ s range (R1) is attributed to interparticle contact resistance, while the peaks spanning 10^−4^–10^−2^ s (R2–R4) are mainly associated with lithium‐ion transport through the cathode–electrolyte interphase (CEI) layer and solid electrolyte interphase (SEI) formed on the lithium anode [49, 50]. The relaxation peak at 10^−2^−10^0^ s (R5, R6) corresponds to the charge‐transfer process at cathode/CEI interface [51, 52], whereas the low‐frequency peak beyond 10^0^ s (R7) predominantly reflects solid‐state lithium diffusion within the cathode particles [53]. At room temperature (25°C), the DRT spectra of SP‐NCM613 show no significant variation in the intensity of the relaxation peaks within the charging voltage window of 3.9–4.4 V (Figure S12b), indicating stable kinetic behavior during the charging process. A slight increase in R7 is observed at higher voltages, which is likely associated with the H2–H3 phase transition of the layered structure. This transition induces a pronounced contraction along the c‐axis, leading to the narrowing of lithium diffusion channels and thereby increasing the resistance related to solid‐state lithium diffusion within the cathode particles [49]. In contrast, during discharge (Figure S12c), the intensity of R7 gradually decreases until approximately 4.0 V and subsequently increases at lower voltages. This behavior could be associated with the sequential H3–H2–H1 phase transitions of the layered structure, where repeated contraction and expansion along the c‐axis modify lithium diffusion pathways and lead to variations in the solid‐state diffusion resistance [54]. The DRT spectra of CP‐NCM613 exhibit higher resistance throughout the charging process compared with SP‐NCM613. Notably, a significant increase in R7 is observed near 4.4 V (Figure S12e), together with enlarged charge‐transfer resistance (R5–R6). Such behavior implies the presence of additional kinetic limitations in CP‐NCM613, which may be related to structural instability or interfacial degradation at high voltages. Moreover, significantly larger and more fluctuating R7 intensity values are also observed during subsequent lithiation (Figure S12f), indicating sluggish lithium diffusion within the cathode particles of CP‐NCM613.

**FIGURE 4:**
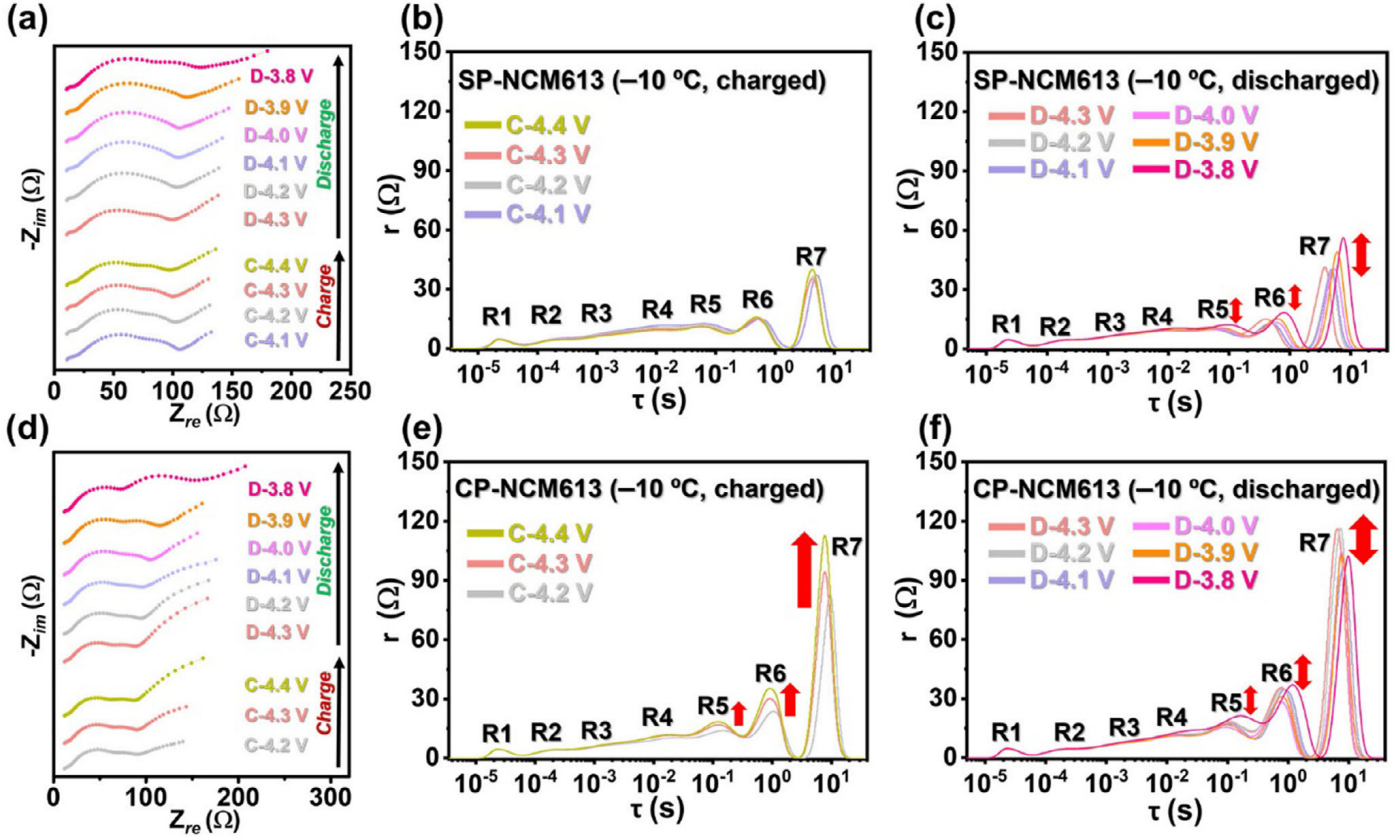
a) In situ Nyquist plots of SP‐NCM613 obtained at preselected potentials during the initial cycle at 0.1 C‐rate and −10°C, with corresponding DRT profiles during b) charging and c) discharging. d) In situ Nyquist plots of CP‐NCM613 obtained under the same conditions, with corresponding DRT profiles during e) charging and f) discharging.

As depicted in Figure 4, these differences become more pronounced under low‐temperature conditions. Consistent with the voltage overshoot observed during the initial charge at −10°C (Figure 3d), electrochemical activation is significantly delayed due to sluggish kinetics. Accordingly, the EIS spectra of SP‐NCM613 (Figure S13a) exhibit elevated resistance in the low‐voltage region (3.8–4.0 V), whereas for CP‐NCM613 (Figure S13b), reliable EIS spectra could not be obtained below 4.1 V owing to insufficient activation. Therefore, the DRT analysis was performed using spectra collected after excluding these inadequately activated regions. Compared to room temperature, the −10°C DRT profiles (Figure 4b,c,e,f) display generally higher resistance contributions and a shift toward longer relaxation times, reflecting aggravated kinetic limitations. Notably, the resistance disparity between SP‐NCM613 and CP‐NCM613 becomes more pronounced under low‐temperature operation than at room temperature, with a particularly significant increase in the R7 component, indicative of severely hindered solid‐state lithium diffusion. Overall, while the R1–R4 components evolve similarly for both samples, the R5–R6 peaks associated with charge‐transfer processes at the cathode/CEI interface increase noticeably. However, the most pronounced divergence occurs in the diffusion‐related R7 component, which exhibits by far the largest difference between the two samples, identifying solid‐state lithium transport as the primary origin of the low‐temperature performance disparity. The larger R5–R6 values observed for CP‐NCM613 further suggest possible degradation of the cathode surface, likely induced by sluggish diffusion kinetics under low‐temperature operation.

Lithium diffusion kinetics critically govern the electrochemical stability of micron‐sized primary particles, as steep concentration gradients can induce localized lattice shear or surface gliding, eventually leading to structural degradation. To quantitatively compare diffusion behavior, galvanostatic intermittent titration technique (GITT) analysis was performed. As shown in Figure S14, both samples exhibit comparable lithium diffusivity during the first cycle at 25°C, with SP‐NCM613 showing slightly higher values. In contrast, the disparity becomes significantly amplified at −10°C, indicating that internal grain boundaries impose a much stronger diffusion limitation under low‐temperature conditions. Considering that sluggish lithium diffusion can generate severe concentration gradients within micron‐sized primary particles, the associated surface deformation was examined by ex‐situ SEM analysis. Upon charging to 4.4 V at 25°C (Figure S15a,b), both cathodes display evident surface gliding features, which can be attributed to mechanical stress generated by lithium concentration gradients within the particles [55−58]. After subsequent discharge to 3.0 V (Figure S15c,d), these features and associated microcracks are largely mitigated, suggesting predominantly reversible deformation behavior, consistent with previous reports [55].

**FIGURE 5:**
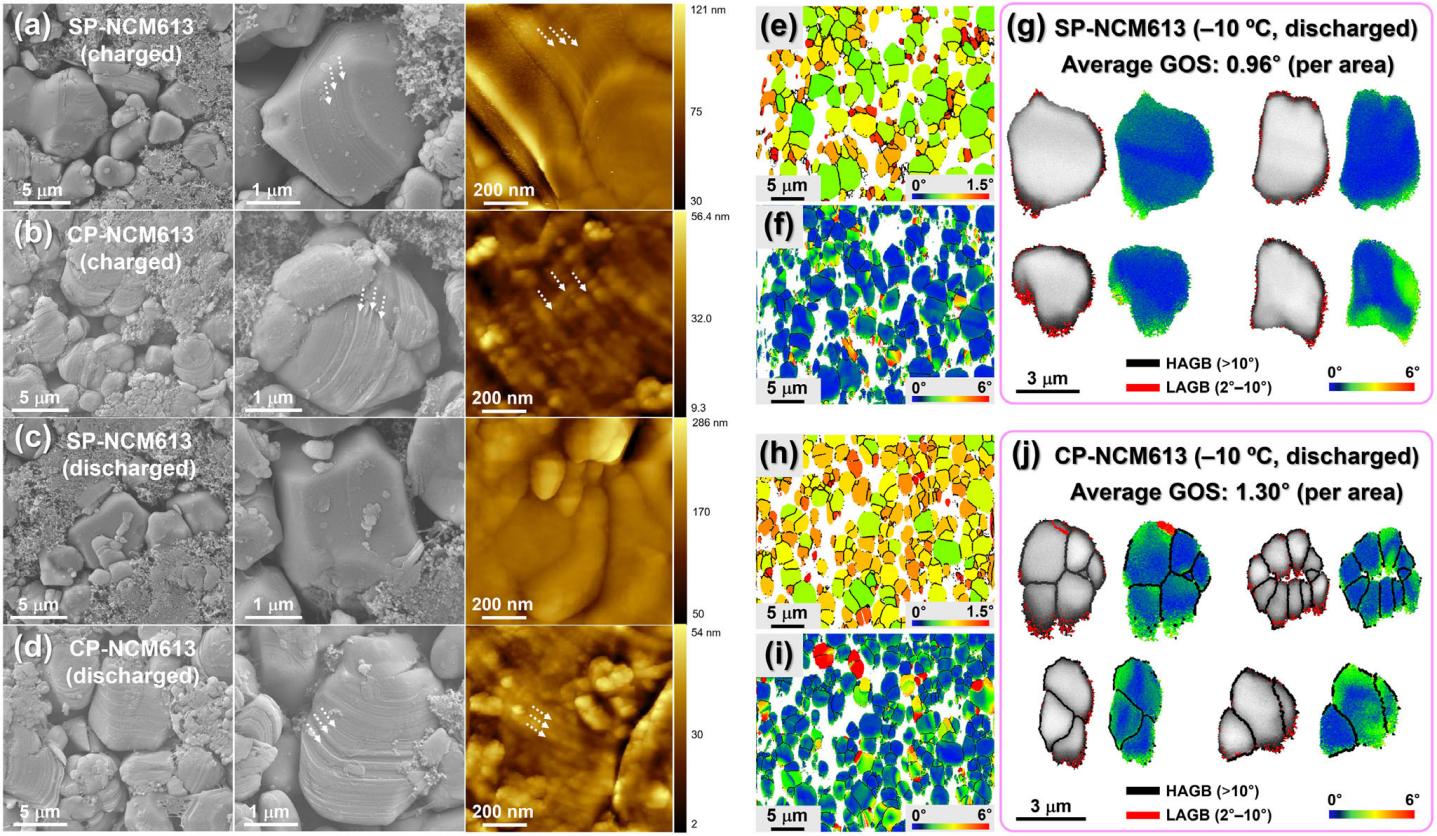
Ex situ SEM and AFM images of a) SP‐NCM613 and b) CP‐NCM613 electrodes after the first full charge to 4.4 V at −10°C. c) SP‐NCM613 and d) CP‐NCM613 electrodes after the first full discharge to 3.0 V at −10°C. EBSD results of SP‐NCM613 and CP‐NCM613 after the first full discharge to 3.0 V at −10°C: e,h) GOS maps of SP‐NCM613 and CP‐NCM613, respectively. f,i) GROD maps of SP‐NCM613 and CP‐NCM613, respectively. g,j) Magnified EBSD maps showing grain boundaries and local orientation deviations within individual particles of SP‐NCM613 and CP‐NCM613, respectively.

However, distinct irreversible structural features emerge under low‐temperature operation, as discussed below. In addition to the ex‐situ SEM images, atomic force microscopy (AFM) provides complementary information on the surface topography. Upon subsequent discharge, SP‐NCM613 (Figure 5c) shows substantial recovery of the gliding features, whereas CP‐NCM613 (Figure 5d) retains a significant portion of the deformation, evidencing irreversible structural damage at low‐temperature. Such persistent gliding behavior originates from severely hindered lithium diffusion imposed by residual internal grain boundaries, which intensifies lithium concentration gradients within the particles [59]. The amplified mechanical stress subsequently promotes surface microcrack formation and parasitic interfacial reactions, ultimately deteriorating electrochemical performance [60]. In light of these observations, the increased charge‐transfer resistances (R5 and R6 in Figure 4e,f) observed in the low‐temperature DRT profiles can be attributed to deteriorated charge‐transfer kinetics at the electrode‐electrolyte interphase caused by surface degradation. These results highlight that diffusion limitations originating from residual grain boundaries can critically influence interfacial reaction kinetics under low‐temperature operation.

Although surface deformation provides important insights into the mechanical degradation of single‐crystal NCM cathodes, the internal crystallographic distortion associated with lithium transport heterogeneity can also critically influence structural stability and degradation behavior. Therefore, cross‐sectional EBSD analysis was performed on the electrodes collected at the fully discharged state after low‐temperature cycling to investigate the internal deformation characteristics. As shown in the Figure 5e−j, the degree of local lattice distortion and orientation deviation was evaluated using grain orientation spread (GOS) and grain reference orientation deviation (GROD) analyses. The average GOS value of SP‐NCM613 was 0.96°, which was lower than that of CP‐NCM613 (1.30°). This result indicates that CP‐NCM613 experienced a higher degree of local crystallographic distortion and more heterogeneous strain accumulation compared with SP‐NCM613 after low‐temperature cycling [61]. Moreover, the GROD maps revealed the spatial distribution of crystallographic distortion within individual particles. In SP‐NCM613 (Figure 5f,g), the orientation deviation was mainly concentrated near the particle surface, which is consistent with previously reported deformation behavior in single‐crystal NCM cathodes, where surface‐localized strain can originate from lithium concentration gradients during electrochemical cycling [9]. In contrast, CP‐NCM613 (Figure 5i,j) exhibited pronounced orientation deviations not only near the particle surface but also around internal grain boundaries. This behavior suggests that the presence of internal grain boundaries may contribute to heterogeneous lithium transport and localized lithium concentration gradients, which could promote strain accumulation near these regions. The accumulated strain may further facilitate local structural deformation and surface gliding during low‐temperature cycling.

**FIGURE 6:**
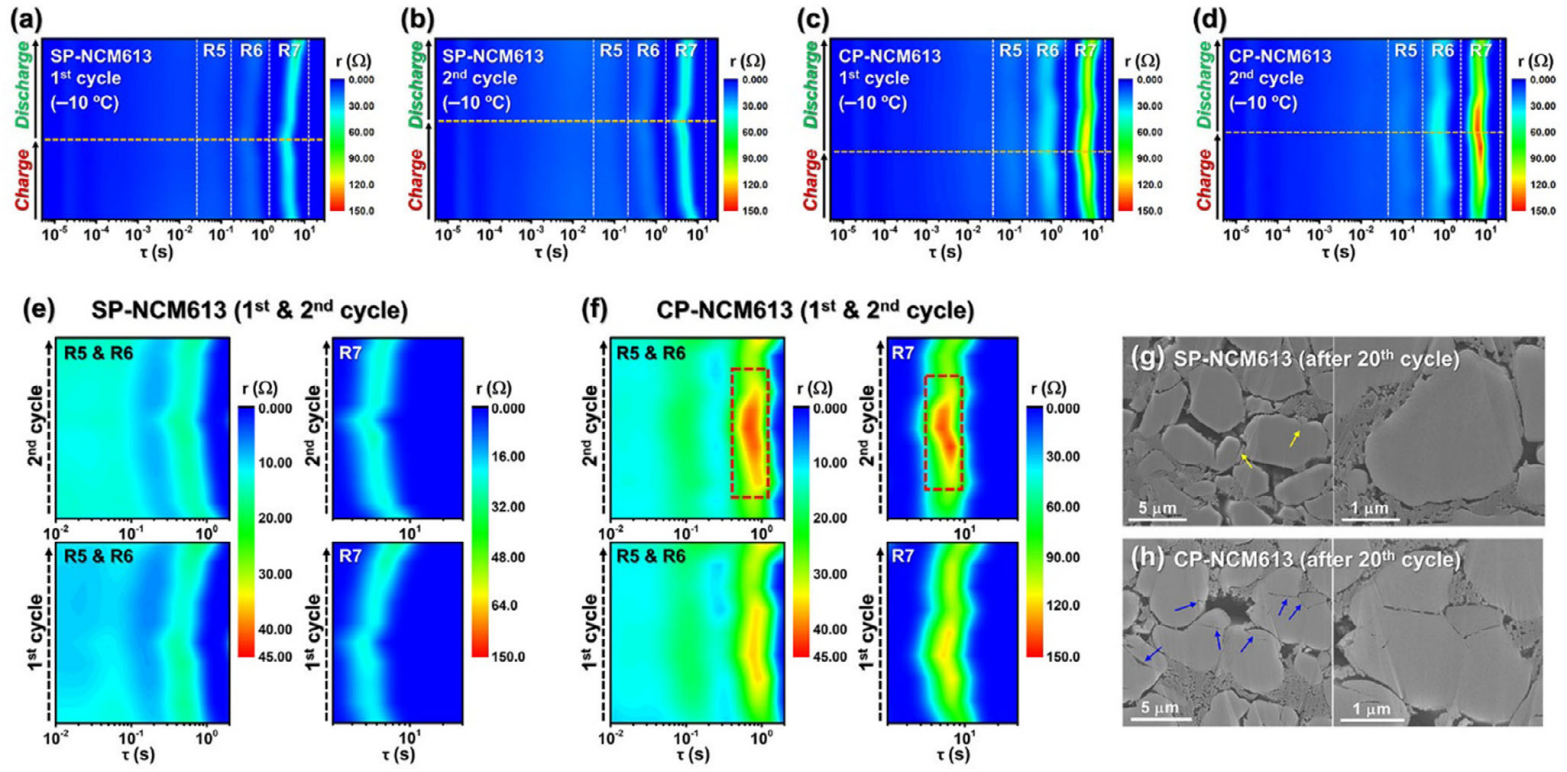
Contour plots of the DRT profiles of SP‐NCM613 and CP‐NCM613 obtained during the first and second cycles at −10°C: a,b) SP‐NCM613 and c,d) CP‐NCM613. Enlarged views of the regions corresponding to R5–R6 and R7 are shown for e) SP‐NCM613 and f) CP‐NCM613. Cross‐sectional SEM images of the electrodes after 20 cycles at −10°C: g) SP‐NCM613 and h) CP‐NCM613. The electrode cross‐sections were prepared using an ion‐milling process.

To further elucidate the impact of surface degradation formed during the first cycle at low‐temperature on the kinetic behavior, in situ EIS and DRT analyses were performed during the second cycle. Owing to the activation process occurring in the initial cycle, the overall impedance in the low‐voltage region was reduced in the second cycle (Figure S16 and S17). However, the resistance at the early stage of charge, particularly in the low‐voltage range of 3.8–3.9 V, remained relatively high. Therefore, this region was excluded from the subsequent DRT analysis. Interestingly, the evolution of resistance during the second cycle exhibits contrasting behaviors between the two cathodes. To enable a more intuitive comparison of the resistance evolution between the first and second cycles for each sample, the DRT plots obtained under low‐temperature operation were further visualized as contour plots (Figure 6a–d). The regions corresponding to R5–R6 and R7 were further enlarged to better highlight their evolution, as shown in Figure 6e for SP‐NCM613 and Figure 6f for CP‐NCM613. Compared to the first cycle, the DRT profiles of SP‐NCM613 (Figure 6e) show no significant variation, maintaining similar resistance values and overall trends. In contrast, CP‐NCM613 (Figure 6f) displays substantially increased charge‐transfer resistances (R5 and R6) relative to the first cycle, along with a pronounced rise in the diffusion‐related resistance (R7), indicating further deterioration of the kinetic properties. The regions showing this degradation are highlighted with red dashed boxes in the figure for clarity. Consistent with the low‐temperature electrochemical results (Figure 3f), where CP‐NCM613 exhibits a rapid capacity decay during the initial ∼20 cycles, these findings suggest that sluggish kinetics at low‐temperature can induce structural degradation and consequent capacity fading from the early stages of cycling. This interpretation is further supported by cross‐sectional SEM images of the electrodes after 20 cycles, prepared via an ion‐milling process (Figure 6g,h). In the case of SP‐NCM613, the particles largely preserve their structural integrity, exhibiting a robust morphology with only minor intergranular cracking (indicated by yellow arrows). In contrast, CP‐NCM613 shows pronounced structural deterioration; despite its quasi‐single‐crystal structure, numerous intergranular cracks (indicated by blue arrows) are observed, along with evident particle fracture, indicating severe mechanical instability. Therefore, it can be understood that grain‐boundary‐induced sluggish kinetics can trigger early‐stage degradation, which in turn leads to increased charge‐transfer resistance and hindered lithium diffusion.

Building on the above observations, where sluggish lithium diffusion at low‐temperature induces structural degradation, both charge‐transfer and diffusion processes can be affected by degradation‐induced artifacts. Thus, decoupling the intrinsic kinetic properties from these effects is essential to gain deeper insight into the degradation behavior. To this end, a temperature‐controlled EIS protocol was employed. First, a formation cycle was conducted identically at room temperature to eliminate the influence of initial activation. The current density was set to a very low rate of 0.05 C to minimize degradation associated with kinetic limitations. After this formation step and a subsequent rest period, impedance spectra were recorded at various temperatures. Detailed experimental procedures and the measurement protocol are provided in Figure S18 and the accompanying Supporting Note. Although the complete elimination of temperature‐induced degradation is challenging, the present protocol substantially suppresses degradation‐driven contributions, enabling a more reliable comparison of intrinsic lithium diffusion kinetics.

**FIGURE 7:**
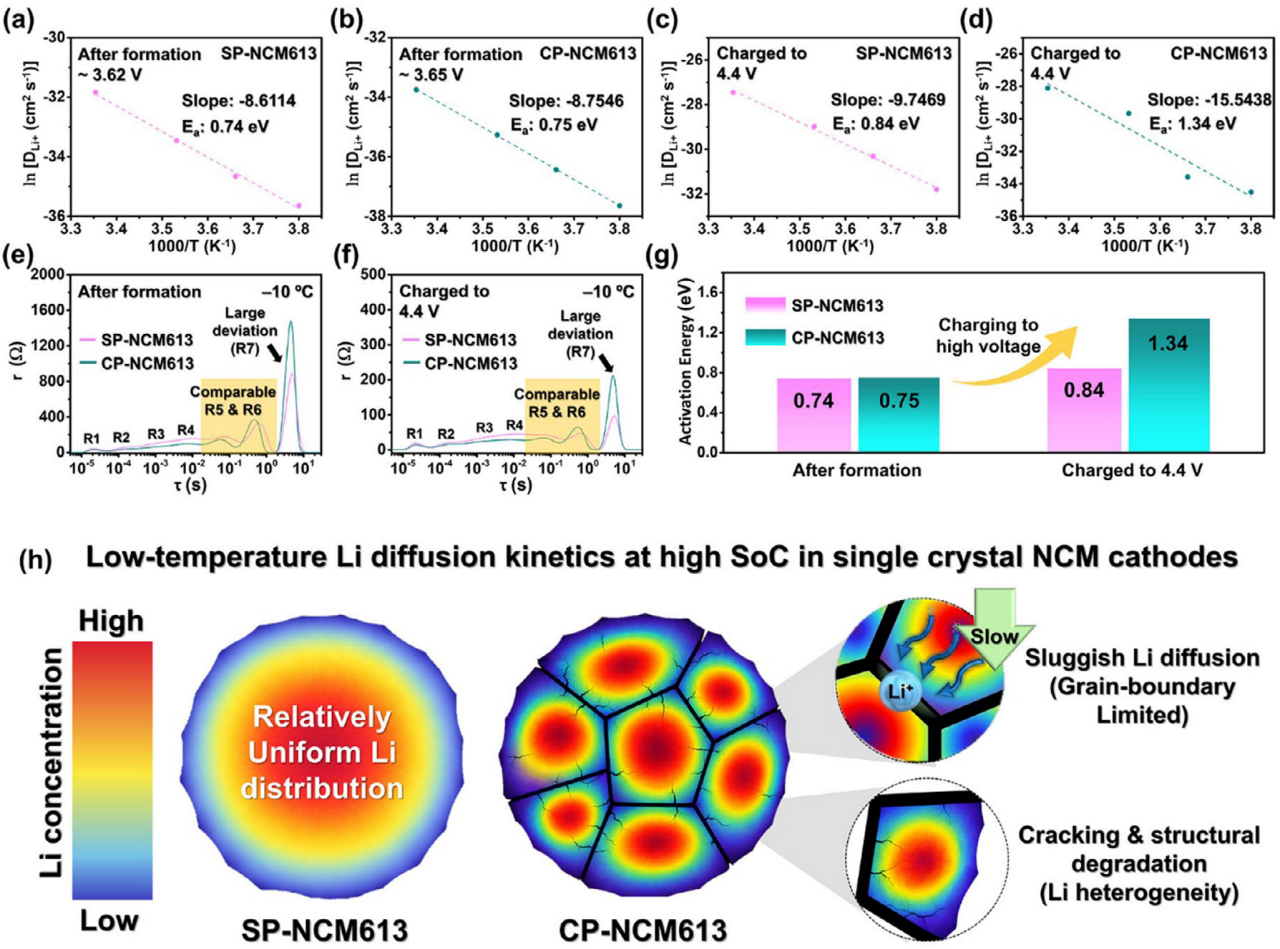
a,b) Arrhenius plots for SP‐NCM613 and CP‐NCM613 cathodes after formation. c,d) Arrhenius plots for SP‐NCM613 and CP‐NCM613 cathodes after charging to 4.4 V. e,f) DRT profiles of the NCM613 cathodes at −10°C after formation and at the 4.4 V charged state, respectively. g) Diffusion activation energy variation of the NCM613 cathodes at different voltage regions. h) Schematic illustration of low‐temperature lithium diffusion kinetics governed by internal grain boundaries.

As illustrated in Figure S18, after the formation cycle the cells were allowed to rest until the voltage stabilized at approximately 3.62 and 3.65 V, respectively. Impedance spectra were then measured at different temperatures under this stabilized state, and the corresponding Nyquist plots are presented in Figure S19a,b. From these Nyquist plots, the Li‐ion diffusion coefficient (D_Li+_) was calculated, and the resulting values are summarized in Figure S19c,d. Using the temperature‐dependent D_Li+_ values, the activation energy for Li‐ion diffusion was determined from Arrhenius plots [62], and the corresponding results are presented in Figure 7a,b. Interestingly, the calculated activation energies of the two cathodes were found to be similar, suggesting that lithium diffusion kinetics influenced by grain boundaries are less sensitive to temperature in the low‐voltage (or low state‐of‐charge, SoC) region. To further examine how the kinetic characteristics evolve with cell voltage, the cells were charged to 4.4 V at room temperature using a low‐current rate of 0.05 C, followed by a resting period until the voltage stabilized. Subsequently, temperature‐dependent EIS measurements were conducted under the same protocol. The corresponding Nyquist plots and the calculated lithium diffusion coefficients are summarized in Figure S20. Based on these results, the activation energies were determined, and the results are presented in Figure 7c,d, revealing a pronounced difference between the two cathodes.

Moreover, DRT profiles derived from the temperature‐controlled EIS data reveal that both cathodes exhibit nearly identical charge‐transfer resistance values (corresponding to R5 and R6) at −10°C, both after the formation cycle and after charging to 4.4 V (Figure 7e,f). In contrast, a clear difference emerges only in R7, which is associated with the lithium diffusion process. Compared with the earlier DRT profiles (Figure 4 and Figure S17), where minor differences were also observed in R5 and R6, the present analysis more clearly isolates the difference in R7. A similar trend is observed in the additional DRT profiles shown in Figure S21, where the pronounced difference again appears predominantly in R7. These behaviors imply that minimizing the influence of kinetic degradation arising during low‐temperature operation enables a clearer assessment of the intrinsic kinetic characteristics of the cathodes. Under such conditions, both materials exhibit comparable charge‐transfer resistance, whereas the dominant difference arises from lithium diffusion, indicating that diffusion kinetics predominantly govern the intrinsic kinetic limitation.

As shown in Figure 7g, the diffusion activation energy of CP‐NCM613 increases sharply at high voltage, whereas SP‐NCM613 maintains a relatively low activation energy. Accordingly, minimizing internal grain boundaries in single‐crystal‐like NCM cathodes is critical for maintaining favorable lithium diffusion kinetics in the high‐voltage region under low‐temperature conditions, enabling a relatively uniform lithium distribution within the particle. In contrast, when internal grain boundaries remain abundant, lithium diffusion becomes increasingly hindered at high voltage as the temperature decreases, leading to heterogeneous lithium distribution, internal stress accumulation, and ultimately crack formation accompanied by structural degradation. The overall mechanism is summarized schematically in Figure 7h, providing important insights into the practical design of single‐crystal NCM cathodes for low‐temperature operation.

## 3 Conclusions

In this study, we systematically elucidate the role of residual internal grain boundaries in governing the low‐temperature electrochemical performance of single‐crystal NCM613 cathodes. Although spray pyrolysis and co‐precipitation derived cathodes exhibit comparable crystal structures, particle sizes, and room‐temperature performance, pronounced disparities emerge under −10°C operation. In situ EIS coupled with DRT analysis reveals that CP‐NCM613 suffers from significantly aggravated diffusion‐related resistance at low‐temperature, which is further supported by GITT results. The morphological analyses confirm that hindered lithium diffusion induces persistent surface gliding and irreversible structural degradation in CP‐NCM613, whereas SP‐NCM613 largely restores its morphology upon discharge. Moreover, the diffusion activation energies of the two samples diverge markedly at high states of charge, indicating that residual internal grain boundaries critically influence lithium diffusion kinetics under low‐temperature and high‐voltage conditions. This understanding provides a useful design guideline for developing structurally robust single‐crystal cathodes capable of maintaining favorable Li transport under high‐voltage and low‐temperature conditions.

## 4 Experimental Section

The single‐crystal NCM613 cathodes investigated in this study were supplied by SK On and synthesized via two distinct, industrially relevant routes. Specifically, SP‐NCM613 and CP‐NCM613 were prepared using spray pyrolysis and co‐precipitation methods, respectively. The precursors obtained from each synthesis route were subsequently subjected to high‐temperature calcination to induce single‐crystallization. The resulting single‐crystal particles were further subjected to sequential Co‐ and W‐based surface modification processes. Detailed synthesis parameters are not disclosed due to confidentiality considerations. Cross‐sectional polishing was performed using a cooling cross section polisher (IB‐19520CCP, Park Systems JEOL; NFEC‐2019‐03‐254558) at the National NanoFab Center (Daejeon, Korea). Scanning electron microscopy (SEM) and electron backscatter diffraction (EBSD) analyses were carried out using a high‐performance dual‐beam focused ion beam system (Helios 5 UX, Thermo Fisher Scientific; NFEC‐2022‐05‐279171) at the National NanoFab Center (Daejeon, Korea). Particle size distribution was measured using a particle size analyzer based on laser diffraction (Malvern Panalytical, Mastersizer 3000). The specific surface area was determined by nitrogen adsorption–desorption measurements using an ASAP 2420 analyzer (Micromeritics, USA). Both analyses were conducted at the Korea Basic Science Institute (KBSI), Jeonju (Equipment code: CJ102). X‐ray diffraction (XRD) data were collected by JP/SmartLab diffractometer manufactured by Rigaku. X‐ray photoelectron spectroscopy (XPS) measurements were performed using a Nexsa XPS system (KBSI, Jeonju; Equipment code: CJ115). Depth profiling was carried out by Ar^+^ monatomic sputtering at an acceleration energy of 3 keV. Specimens for TEM analysis were prepared using a focused ion beam (FIB; Helios 5 UX, Thermo Fisher Scientific, KBSI, Daejeon; Equipment code: EM01). High‐resolution TEM was conducted using a JEOL JEM‐2100F (KBSI, Daejeon; Equipment code: AE12) operating at 200 kV, with imaging performed under low‐dose conditions to minimize beam‐induced artifacts. Atomic force microscopy (AFM) measurements were performed using a NX7 system (Park Systems, Korea). An ElectriMulti75‐G cantilever (nominal spring constant ∼3 N/m, resonance frequency ∼75 kHz) was used, and the measurements were conducted in non‐contact mode. All AFM measurements were carried out under ambient conditions (25°C, relative humidity ∼10%). Images were acquired at a scan rate of 0.2 Hz with a resolution of 256 × 256 pixels over a scan area of 1 × 1 µm^2^. The scan direction was from bottom to top. Detailed information pertaining to the electrochemical measurements of the prepared samples is provided in Supporting Information.

## Author Contributions

**Yeong Beom Kim**: data curation, formal analysis, investigation, Writing – original draft. **Yun Chan Kang**: conceptualization, supervision, writing – review and editing. **Jihong Park**: data curation, formal analysis, writing – original draft, investigation. **Jaewon Lee**: data curation, formal analysis, investigation, writing – original draft. **Jihoon Choi**: conceptualization, data curation, formal analysis, writing – review and editing, supervision. **Jihye Jang**: data curation, investigation, formal analysis. **Gi Dae Park**: conceptualization, data curation, formal analysis, investigation, writing – original draft, writing – review and editing, supervision. **Seunghyeon Chae**: data curation, formal analysis. **Gigap Han**: data curation, investigation, formal analysis. **Sang Mun Jeong**: supervision, writing – review and editing. **Dohyung Kim**: investigation, formal analysis, data curation. **Hyeongi Song**: data curation, investigation, formal analysis.

## Conflicts of Interest

The authors declare no conflicts of interest.

## Supporting information




**Supporting File**: advs77448‐sup‐0001‐SuppMat.docx.

## Data Availability

The data that support the findings of this study are available from the corresponding author upon reasonable request.
