# Supplementary material for: Synthesis‐Dependent Grain Boundary Density Governs Low‐Temperature Lithium Diffusion in Single‐Crystal NCM Cathodes
Source: Adv Sci (Weinh). 2026 Aug 31:e77448. Online ahead of print. doi: 10.1002/advs.77448 (PMC13528682; doi:10.1002/advs.77448)
Supplement: Supplementary file 1 [file ADVS-9999-e77448-s001.docx]

((Supporting Information can be included here using this template))

Copyright WILEY-VCH Verlag GmbH & Co. KGaA, 69469 Weinheim, Germany, 2013.

Supporting Information

**Synthesis-dependent grain boundary density governs low-temperature lithium diffusion in single-crystal NCM cathodes**

*Yeong Beom Kim*^+^*, Jaewon Lee*^+^*, Jihong Park*^+^*, Seunghyeon Chae, Dohyung Kim, Gigap Han, Hyeongi Song, Jihye Jang, Jihoon Choi***, Sang Mun Jeong*, Yun Chan Kang*, Gi Dae Park**

Y.B. Kim, Prof. Y.C. Kang*

Department of Materials Science and Engineering

Korea University

Anam-dong, Seongbuk-gu

Seoul 136-713, Republic of Korea

E-mail: yckang@korea.ac.kr

J. Lee, J. Park, S. Chae, Prof. D. Kim, Prof. G.D. Park*

Department of Advanced Materials Engineering

Chungbuk National University

Chungdae-ro 1, Seowon-gu, Cheongju

Chungbuk 28644, Republic of Korea

E-mail: gdpark@chungbuk.ac.kr

J. Lee, J. Park, Prof. G.D. Park*

Department of Urban, Energy, and Environmental Engineering

Chungbuk National University

Chungdae-ro 1, Seowon-gu, Cheongju

Chungbuk 28644, Republic of Korea

E-mail: gdpark@chungbuk.ac.kr

G. Han, H. Song, J. Jang, J. Choi*

SK On R&D Center

Daejeon, Republic of Korea

E-mail: jihoonchoi@sk.com

Prof. S.M. Jeong*

Department of Chemical Engineering

Chungbuk National University

Chungdae-ro 1, Seowon-gu, Cheongju

Chungbuk 28644, Republic of Korea

E-mail: smjeong@chungbuk.ac.kr

Prof. S.M. Jeong*, Prof. G.D. Park*

Advanced Energy Research Institute

Chungbuk National University

Chungdae-ro 1, Seowon-gu, Cheongju

Chungbuk 28644, Republic of Korea

E-mail: gdpark@chungbuk.ac.kr

**Keywords:** Mid-nickel NCM cathodes, Single-crystal cathodes, Spray pyrolysis, Grain boundary effects, Low-temperature lithium diffusion

^+^ These authors contributed equally to this work.

**Electrochemical measurements**

All electrochemical properties were investigated using coin-type cells (CR2032), which were assembled in an argon-filled glove box to ensure an inert environment. All electrodes were punched to a diameter of 14 mm. To prepare the cathode electrode, a mixture of single-crystal NCM613 powder, Super P (Timcal), and polyvinylidene fluoride (PVDF, Kureha KF9700) was combined in a weight ratio of 90: 5: 5. This mixture was then dispersed in NMP solvent and coated onto the Al foil. The loading mass of NCM613 cathode was 12 mg cm^−2^. For full-cell tests, commercial graphite was employed as the anode material. The graphite electrodes were fabricated using the same procedure as the cathode electrodes, with an areal loading of approximately 6.7 mg cm^−2^. The electrolyte consisted of 1.15 M LiPF_6_ dissolved in ethylene carbonate/ethyl methyl carbonate (EC/EMC, v/v = 3:7, Enchem) with 2 wt% vinylene carbonate (VC) as an additive, and the total electrolyte volume used for each cell was 150 μL. In-situ electrochemical impedance spectroscopy (EIS, ZIVE SP1) analysis was performed at preselected potentials during the charge and discharge process at a current density of 0.1 C-rate. The EIS measurements of the electrode were performed over a frequency range of 0.001 Hz–100 kHz. The distribution of relaxation time (DRT) transformation was performed using MATLAB software with all parameters kept consistent throughout the calculations to ensure the reliability of the DRT results. The chemical diffusion coefficients of lithium ions were determined using the galvanostatic intermittent titration technique (GITT).^[S1]^ The cells were charged and discharged using 10 min current pulses at 0.2 C at 25 °C and 0.1 C at −10 °C, followed by 20 min relaxation periods under open-circuit conditions.

**Figure S1.** Characterization of the precursor materials: (a,b) SEM images of SP-precursor and CP-precursor, respectively; (c) particle-size distributions; and (d) XRD patterns. The peaks marked with asterisks correspond to secondary hydroxide phases, such as Co(OH)_2_.

As shown in the SEM images, both precursors exhibited spherical morphologies. However, noticeable differences were observed in their internal structures, particle size distributions, and average particle sizes. Specifically, the spray pyrolysis-derived precursor exhibited a hollow spherical morphology, whereas the co-precipitation-derived precursor possessed a dense spherical structure, as highlighted in the SEM inset images. In addition, particle size distribution analysis revealed that the co-precipitation-derived precursor exhibited a relatively narrow particle size distribution with a uniform average particle size of approximately 4.1 μm, whereas the spray pyrolysis-derived precursor showed a broader particle size distribution. These observations reflect the intrinsic particle formation mechanisms of the two synthesis routes.^[S2,S3]^

The XRD patterns also revealed clear differences in the crystallographic characteristics of the precursors. The spray pyrolysis-derived precursor, synthesized directly from a mixed solution containing Li and transition metal salts, exhibited broad diffraction peaks with relatively low crystallinity. This behavior is attributed to the extremely short residence time during the spray pyrolysis process, which limits crystal growth and crystallization, resulting in the coexistence of partially crystallized transition metal oxide-based rock-salt phases and layered NCM phases. In contrast, the co-precipitation-derived precursor exhibited relatively sharp diffraction peaks characteristic of a well-crystallized hydroxide phase, indicating that sufficient grain growth and crystallization occurred during the co-precipitation process, consistent with previous reports on co-precipitated NCM hydroxide precursors.^[S4]^

**Figure S2.** Particle-size distribution of a) SP-NCM613 and b) CP-NCM613. N_2_ gas adsorption isotherms of c) SP-NCM613 and d) CP-NCM613.

**Figure S3.** Cross-sectional SEM images of the SP-NCM613 and CP-NCM613 electrodes prepared by ion milling: a) SP-NCM613 and b) CP-NCM613.

**Figure S4.** Cross-sectional SEM images, band contrast EBSD maps, and crystallographic orientation maps of a**–**c) SP-NCM613 and e–g) CP-NCM613, along with elemental mapping images of d) SP-NCM613 and h) CP-NCM613.

**Figure S5.** XPS depth profiles of Co 2p for a) SP-NCM613 and b) CP-NCM613; W 4f for c) SP-NCM613 and d) CP-NCM613, obtained after sputtering for 240 and 480 s.

**Figure S6.** a) STEM-HAADF image of SP-NCM613 and b) the corresponding EELS line profiles acquired along the scanning path indicated in a) for SP-NCM613. c) STEM-HAADF image of CP-NCM613 and d) the corresponding EELS line profiles acquired along the scanning path indicated in c) for CP-NCM613.

**Figure S7.** Half-cell electrochemical performance of the spray pyrolysis-derived NCM613 cathodes at 25 °C: Initial charge–discharge voltage profiles at 0.1 C for (a) the pristine NCM613 cathode without surface modification and (b) the Co surface-modified NCM613 cathode.

**Figure S8.** dQ/dV curves of a) SP-NCM613 and b) CP-NCM613 during cycling at 25 °C, 0.3 C-rate.

**Figure S9.** dQ/dV curves of a) SP-NCM613 and b) CP-NCM613 during cycling at −10 °C, 0.3 C-rate.

**Figure S10.** Electrochemical performance of commercial graphite anode half cells evaluated at different temperatures: a) initial charge-discharge voltage profile measured at 25 °C and 0.035 A g^−1^; b) initial charge-discharge voltage profile measured at −10 °C with current densities of 0.035 A g^−1^ and 0.0175 A g^−1^.

**Figure S11.** Electrochemical performance of SP-NCM613/graphite full cells evaluated at different temperatures: a) initial charge-discharge voltage profile measured at 25 °C and 0.1 C, b) cycling performance at 25 °C with a current rate of 1.0 C, c) initial charge-discharge voltage profile measured at −10 °C and 0.1 C; d) cycling performance at −10 °C with a current rate of 0.3 C. All cycling tests were conducted after two initial activation cycles at 0.1 C.

**Figure S12.** a) In-situ Nyquist plots of **SP-NCM613** obtained at preselected potentials during the initial cycle at **0.1 C-rate and 25 °C**, with corresponding **DRT profiles** during b) **charging** and c) **discharging**. d) In-situ Nyquist plots of **CP-NCM613** obtained under the same conditions, with corresponding **DRT profiles** during e) **charging** and f) **discharging**.

**Figure S13.** In-situ Nyquist plots of a) **SP-NCM613 and b) CP-NCM613** at preselected potentials during the initial cycle at **0.1 C-rate and −10 °C.**

**Figure S14.** a) GITT curves of SP-NCM613 and CP-NCM613 during the initial cycle at 0.2 C and 25 °C. Lithium-ion diffusion coefficients determined by GITT during the initial b) charge and c) discharge at 0.2 C and 25 °C. d) GITT profiles during the initial cycle at 0.1 C and −10 °C, and the corresponding diffusion coefficients during e) charge and f) discharge.

The lithium-ion diffusion coefficients (D_Li+_) were calculated by GITT analysis. The GITT tests were conducted at a current density of 0.2 or 0.1 C, featuring a pulse time of 10 minutes and a relaxation time of 20 minutes. The D_Li+_ values were calculated using the equation below:

D_Li+_ = 4/πτ (*m_B_V_M_*/*M_B_S*)^2^∙(∆*E_s_*/∆*E_τ_*)^2^

The necessary parameter values are documented in a prior study.^[S1]^

**Figure S15.** Ex-situ SEM images of a) SP-NCM613 and b) CP-NCM613 electrodes after the first full charge to 4.4 V at 25 °C. c) SP-NCM613 and d) CP-NCM613 electrodes after the first full discharge to 3.0 V at 25 °C.

**Figure S16.** In-situ Nyquist plots of a) **SP-NCM613 and b) CP-NCM613** at preselected potentials during the second cycle at **0.1 C-rate and −10 °C.**

**Figure S17.** a) In-situ Nyquist plots of **SP-NCM613** obtained at preselected potentials during the second cycle at **0.1 C-rate and −10 °C**, with corresponding **DRT profiles** during b) **charging** and c) **discharging**. d) In-situ Nyquist plots of **CP-NCM613** obtained under the same conditions, with corresponding **DRT profiles** during e) **charging** and f) **discharging**.

**Figure S18.** Voltage profiles as a function of time during the first formation cycle for a) SP-NCM613 and b) CP-NCM613.

The initial formation cycle was carried out at 25 °C using a slow current rate of 0.05 C in order to minimize degradation-related effects. The charging process up to 4.4 V was performed in CC–CV mode, followed by a CV step at 4.4 V until the current decreased to 0.01 C. Subsequently, the cell was discharged to 3.0 V at 0.05 C. After completing the formation cycle, the cells were kept under open-circuit conditions at 25 °C for 2 h to minimize relaxation effects associated with temperature variation and to stabilize the electrochemical state. During this relaxation period, the cell voltage gradually stabilized and reached a saturated value of approximately ~3.62 and ~3.65 V for SP-NCM613 and CP-NCM613, respectively. For impedance measurements at different temperatures, the environmental temperature was adjusted to the target value. To ensure thermal equilibration of the cells, an additional 3 h rest period was applied at each temperature prior to the measurement. After this stabilization step, EIS was conducted at the corresponding temperature.

**Figure S19.** Nyquist plots obtained from temperature-controlled EIS of a) SP-NCM613 and b) CP-NCM613 measured after the formation cycle. Temperature-dependent lithium diffusion coefficients of c) SP-NCM613 and d) CP-NCM613 calculated from the impedance spectra.

The lithium-ion diffusion coefficient (D_Li+_) was calculated from the linear Warburg region of the $z^{'}$ vs $\omega^{-1/2}$plot, using the following equation:

D_Li+_ = 0.5 (*RT*/*Sn*^2^*F*^2^*C*σ)^2^

The parameter values are explained in the previous study.^[S5]^

**Figure S20.** Nyquist plots of a) SP-NCM613 and b) CP-NCM613 measured at different temperatures after subsequent charging to 4.4 V following the formation cycle. Temperature-dependent lithium diffusion coefficients of c) SP-NCM613 and d) CP-NCM613 derived from the impedance spectra.

**Figure S21.** DRT profiles derived from temperature-controlled EIS measurements of NCM613 cathodes: a) SP-NCM613 and b) CP-NCM613 measured after formation; c) SP-NCM613 and d) CP-NCM613 measured after charging to 4.4 V.

**Table S1.** Structural parameters obtained from the Rietveld refinement of XRD patterns for SP-NCM613 and CP-NCM613.

|  | SP-NCM613 | CP-NCM613 |
| --- | --- | --- |
| a (Å) | 2.87799 (7) | 2.87899 (4) |
| c (Å) | 14.2522 (3) | 14.2543 (2) |
| Vol. (Å^3^) | 102.23 (5) | 102.37 (1) |
| R_p_ (%) | 3.87 | 2.20 |
| R_wp_ (%) | 2.53 | 2.99 |
| Li/Ni mixing (%) | 2.50 | 2.80 |
| I_(003)_/I_(104)_ | 1.89 | 1.75 |

**Table S2.** ICP-OES results of SP-precursor and CP-precursor.

| **Sample** | **Li/TM Ratio** | **Ni / (Ni+Co+Mn)** | **Co / (Ni+Co+Mn)** | **Mn / (Ni+Co+Mn)** | **W [wtppm]** |
| --- | --- | --- | --- | --- | --- |
| SP-precursor | 1.07 | 0.597 | 0.101 | 0.302 | <50 |
| CP-precursor | - | 0.603 | 0.102 | 0.294 | <50 |

| **Sample** | **Li/TM Ratio** | **Ni / (Ni+Co+Mn)** | **Co / (Ni+Co+Mn)** | **Mn / (Ni+Co+Mn)** | **W [wtppm]** |
| --- | --- | --- | --- | --- | --- |
| SP-NCM613 | 1.02 | 0.589 | 0.124 | 0.288 | 4160 |
| CP-NCM613 | 1.01 | 0.591 | 0.122 | 0.287 | 4030 |

**Table S3.** ICP-OES results of SP-NCM613 and CP-NCM613 cathodes.

**Table S4.** Comparison of electrochemical evaluation conditions and performances of recently reported single-crystal NCM cathodes.

| Material | Parameter | Value |
| --- | --- | --- |
| SP-NCM613  (This work) | Cathode loading mass  Primary particle size  Operating voltage  Initial discharge capacity at 0.1 C  1 C capacity retention  Modification  Evaluation temperature | 12 mg cm^−2^  D_50_: 4.31 μm  3.0−4.4 V  193.5 mA h g^−1^  94.8% (200cycles)  Co (~2.5 wt%), W (~0.5 wt%) surface modification  −10 °C ~ 25 °C |
| LiNi_0.6_Co_0.2_Mn_0.2_O_2_^[S6]^ | Cathode loading mass  Primary particle size  Operating voltage  Initial discharge capacity at 0.1 C  1 C capacity retention  Modification  Evaluation temperature | 5.0−7.0 mg cm^−2^  D_50_: 2.42 μm  2.7−4.3 V  185.1 mA h g^−1^  93.2% (200 cycles)  -  25 °C |
| LiNi_0.6_Co_0.2_Mn_0.2_O_2_^[S7]^ | Cathode loading mass  Primary particle size  Operating voltage  Initial discharge capacity at 0.1 C  1 C capacity retention  Modification  Evaluation temperature | 4.42 mg cm^−2^  ~4 μm  2.75−4.25 V  162.0 mA h g^−1^  84.5% (200 cycles)  MoO_3_/Li_2_MoO_4_ coated  25 °C |
| LiNi_0.6_Co_0.1_Mn_0.3_O_2_^[S8]^ | Cathode loading mass  Primary particle size  Operating voltage  Initial discharge capacity at 0.1 C  1 C capacity retention  Modification  Evaluation temperature | ~6 mg cm^−2^  D_50_: 3.75 μm  2.5−4.4 V  205.0 mA h g^−1^  86.8% (1000 cycles)  -  25 °C |
| LiNi_0.7_Co_0.15_Mn_0.15_O_2_^[S9]^ | Cathode loading mass  Primary particle size  Operating voltage  Initial discharge capacity at 0.1 C  1 C capacity retention  Modification  Evaluation temperature | 9.5 ± 0.01 mg cm^−2^  D_50_: 3.27 μm  2.7−4.3 V  ~170.0 mA h g^−1^  83.8% (200 cycles)  -  30 °C |
| LiNi_0.874_Co_0.09_Mn_0.03_Al_0.003_Zr_0.003_O_2_^[S10]^ | Cathode loading mass  Primary particle size  Operating voltage  Initial discharge capacity at 0.1 C  0.5 C capacity retention  Modification  Evaluation temperature | 8.5 ± 0.15 mg cm^−2^  ~ 4.0 μm  2.75−4.6 V  221.6 mA h g^−1^  69.4% (150 cycles)  Al/Zr 0.3 mol% each  25 °C |
| LiNi_0.83_Co_0.11_Mn_0.06_O_2_^[S11]^ | Cathode loading mass  Primary particle size  Operating voltage  Initial discharge capacity at 0.1 C  1 C capacity retention  Modification  Evaluation temperature | 4 mg cm^−2^  3.0−5.0 μm  2.7−4.3 V  193.8 mA h g^−1^  95.4% (150 cycles)  TiBO_3_ coated  25 °C |
| LiNi_0.9_Co_0.05_Mn_0.05_O_2_^[S12]^ | Cathode loading mass  Primary particle size  Operating voltage  Initial discharge capacity at 0.1 C  1 C capacity retention  Modification  Evaluation temperature | 1.7~2.5 mg cm^−2^  3.5 μm  2.7−4.5 V  229.4 mA h g^−1^  94.1% (100 cycles)  In-doped and Li_3_InO_3_ coated  25 °C |

**Supplementary References**

S1. A. V. Ivanishchev, S.-H. Lee, J.-J. Kim, I. A. Ivanishcheva, S.-C. Nam, J.-H. Song, “Li-Ion Diffusion Characteristics of Surface Modified Ni-Rich NCM Cathode Material,” *J. Electroanal. Chem.* 932 (2023): 117242.

S2. S. Tsai, Y. Song, C. Tsai, C. Yang, W. Chiu, H. Lin, “Ultrasonic Spray Pyrolysis for Nanoparticles Synthesis,” *J. Mater. Sci.* 39 (2004): 3647-3657.

S3. J. Chen, S. Feng, J. Deng, Y. Zhou, “Application of Precursor with Ultra-Small Particle Size and Uniform Particle Distribution for Ultra-High Nickel Single-Crystal Cathode Materials by Coprecipitation Method,” *J. Colloid Interface Sci.* 679 (2025): 798-810.

S4. X. Xu, H. Huo, J. Jian, L. Wang, H. Zhu, S. Xu, X. He, G. Yin, C. Du, X. Sun, “Radially Oriented Single‐Crystal Primary Nanosheets Enable Ultrahigh Rate and Cycling Properties of LiNi_0.8_Co_0.1_Mn_0.1_O_2_ Cathode Material for Lithium‐Ion Batteries,” *Adv. Energy Mater.* 9 (2019): 1803963.

S5. J. Zhang, J. Lin, Y. Zeng, Y. Zhang, H. Guo, “Morphological and Structural Evolution of MnO@C Anode and Its Application in Lithium-Ion Capacitors,” *ACS Appl. Energy Mater.* 2 (2019): 8345-8358.

S6. X. Zhao, Q. Ruan, Y. Li, B. Li, X. Yan, X. Zhang, H. Chen, J. Fan, “A Modification Strategy for Synthesizing Single-Crystal LiNi_0.6_Co_0.2_Mn_0.2_O_2_ with Superior Electrochemical Performance,” *Electrochim. Acta* 526 (2025): 146225.

S7. Y. Sun, Z. Li, J. Lu, M. Zhou, S. Liu, L. Qiao, X. Hu, “Functionalized Water Washing Engineering to Improve the Electrochemical Performance of Single-Crystal Ni-Rich Cathode for Lithium-Ion Battery,” *J. Power Sources* 674 (2026): 239775.

S8. B. Heo, M. Kim, C. Hwang, H. W. Kim, M. Pin, B. T. Na, J. B. Lee, C.-u. Bak, J. Y. Cheong, S.-H. Yu, “Interfacial Stability Enhancement in Single-Crystal NCM Cathodes Through Electronic Structure Optimization,” *Mater. Today* 90 (2025): 323-333.

S9. H.-H. Ryu, S.-B. Lee, C. S. Yoon, Y.-K. Sun, “Morphology-Dependent Battery Performance of Ni-Rich Layered Cathodes: Single-Crystal Versus Refined Polycrystal,” *ACS Energy Lett.* 7 (2022): 3072-3079.

S10. X. Ou, T. Liu, W. Zhong, X. Fan, X. Guo, X. Huang, L. Cao, J. Hu, B. Zhang, Y. S. Chu, “Enabling High Energy Lithium Metal Batteries via Single-Crystal Ni-Rich Cathode Material Co-Doping Strategy,” *Nat. Commun.* 13 (2022): 2319.

S11. C. Zeng, R. Zheng, F. Fan, X. Wang, G. Tian, S. Liu, P. Liu, C. Wang, S. Wang, C. Shu, “Phase Compatible Surface Engineering to Boost the Cycling Stability of Single-Crystalline Ni-Rich Cathode for High Energy Density Lithium-Ion Batteries,” *Energy Storage Mater.* 72 (2024): 103788.

S12. H. Zhang, H. Yu, E. Zhang, Q. Cheng, Q. Cheng, H. Jiang, “One-Step Surface-to-Bulk Modification of Single-Crystalline Ni-Rich Co-Poor Cathodes for High-Rate and Long-Life Li-Ion Batteries at High-Voltage Operations,” *Chem. Eng. J.* 498 (2024): 155617.
